# In vivo investigation on bio-markers of perimenopausal panic disorder and catgut embedding acupoints mechanism

**DOI:** 10.1097/MD.0000000000019909

**Published:** 2020-05-08

**Authors:** Guizhen Chen, Xue Wang, Shuo Zhang, Xiaokang Xu, Junquan Liang, Yunxiang Xu

**Affiliations:** a The Bao’an District TCM Hospital, The Affiliated Hospital of Guangzhou University of Chinese Medicine, Shenzhen; b Clinical Medical College of Acupuncture, Moxibustion and Rehabilitation, Guangzhou University of Chinese Medicine, Guangzhou, China.

**Keywords:** proton magnetic resonance spectroscopy, acupoint catgut embedding, functional magnetic resonance imaging, GABA-Glutamate, perimenopausal panic disorder

## Abstract

**Background:**

Panic disorder (PD), defined by repeated and unexpected panic attacks, severely affects patients’ living quality and social function. Perimenopausal women are high-risk group of PD and suffer greatly from it. Modern medicine therapies for this disorder have many side reactions and poor effects, so nonpharmacological modality is an urgent need. Although acupoint catgut embedding is widely used in clinical practice, there is no persuasive evidence of its effect for perimenopausal PD. The aim of this study is to investigate the effectiveness and safety of acupoint catgut embedding for perimenopausal PD and to elucidate the correlations among brain neural activation, bio-markers (amino acids) and clinical outcomes with radiographic evidence, thus to explore its neural mechanism.

**Methods:**

The parallel designed, exploratory randomized controlled trial will include 70 outpatients with perimenopausal PD recruited from two hospitals of Chinese Medicine. These subjects will be randomly allocated to an intervention group (Group Embedding) and a control group (Group Medication) in a 1:1 ratio. The subjects in the intervention group will receive acupoint catgut embedding treatment two weeks a time in the following predefined acupuncture points: Shenshu (BL23), Sanyinjiao (SP6), Guanyuan (RN4), Ganshu (BL18), Zusanli (ST36) and Pishu (BL20). The included women of the control group will take 0.4 mg Alprazolam tablet orally, 1 tablet a time, 3 times a day. There is a study period of 3 months and a follow-up period of 1 month for each group. The primary outcomes will be the following therapeutic indexes: the frequency of panic attack, Panic Disorder Severity Score (PDSS), and Panic-associated Symptoms Score (PASS) during the observation period and follow-up period. The changes in Hamilton Anxiety Scale (HAMA) Score and Symptom Checklist 90 (SCL-90) Score will also be compared between these two groups. Additionally, functional magnetic resonance imaging (fMRI) and proton magnetic resonance spectroscopy (1H-MRS) scans will be done before and after the observation period to show cranial neuroimaging changes.

**Discussion:**

We present a study design and rationale to explore the effectiveness and neural mechanism of acupoint catgut embedding for perimenopausal PD. There are still several factors restrict our research such as no unified standard of diagnostic criteria and curative effect evaluation.

**Trial registration:**

Chinese Clinical Trial Registry, ChiCTR-INR-16009724, registered in November 2016.

## 1 Introduction

The definition of panic disorder (PD) is repeated and unexpected panic attacks.^[1]^ It is common in the general population with a lifetime prevalence of 1.6% to 2.2% and has a high recurrence rate.^[2]^ The main features of PD are excessive and persistent fear and related behavioral disturbances. Attacks are usually accompanied by tachypnea, dizziness, palpitation, tremor, and sweating. Because of PD's sudden onset and a strong sense of impending death, this disease severely affects patients’ living quality and social function, bringing a heavy financial burden on families and society.^[3]^ Perimenopausal women are the high-risk group of PD. They are at a special time when disease intervention is particularly important.

Modern medicine therapies towards perimenopausal PD, such as hormone replacement therapy, rnon-hormonal medication therapy, and cognitive behavioral therapy have many flaws, like the variety of side effects, high recurrence rate, drug dependence, and so on.^[4]^ Clinical medications such as Selective Serotonin Reuptake Inhibitors (SSRI), serotonin-norepinephrine reuptake inhibitors (SNRI), tricyclic antidepressants (TCA), and benzodiazepines (BDZ) are efficacious in the treatment of PD. However, approximately 20% to 40% of PD patients do not fully respond to pharmacotherapy.^[5,6]^ As the “gold standard” of psychotherapy for it, cognitive behavioral therapy (CBT) still does not increase the cure rate.^[7]^ Recently, therapy combining drugs and CBT has become a noticeable research field of PD treatment. It shows great clinical effects in short terms, but still fails to fill this gap. Moreover, 25% to 50% of patients relapse within 6 months after drug discontinuation. Up to 50% of patients suffer from residual panic phobic symptoms after treatment. And more than 30% of patients are still afflicted with a full-blown disorder after 3 to 6 years.^[8]^ From a clinical point of view, a strong need for effective, fast acting and tolerable therapeutic treatments for PD still exists.^[5]^ Therefore, investigating the pathogenesis and seeking for valid curative measures are quite essential to perimenopausal patients, for both their body and mind health care.

Studies have reported that in patients with an anxiety disorder, increased excitatory neurotransmission by glutamate or decreased inhibitory signaling by Gamma-aminobutyric acid (GABA) could be the reasons of increased activity in the emotion-processing brain regions.^[9]^ The drop of inhibitory GABA, leading to an imbalance of Glutamate-GABA, is known as the basic cause of PD.^[10,11]^ Perimenopausal symptoms are related to disruptions to nervous and endocrine systems. The metabolic disorder of amino acid could cause the excitatory and inhibitory imbalance of brain function.^[12]^ In previous research, ovariectomized (OVX) rats were used as models of female climacteric state. OVX caused the elevations of both essential and non-essential amino acids in blood plasma, creating outstanding disruptions in amino acid metabolism. But whether or not that perimenopausal PD patient's neural activity status and neural conductive mechanism are related to the drop of inhibitory GABA still remains unclear. In addition, acupoint catgut embedding restored all the OVX-induced changes except for total cholesterol in plasma. Therefore, this therapy has been proved to modulate amino acid closely related to emotion regulation of OVX rats.^[13]^

Nowadays, acupoint catgut embedding is widely applied in the clinical practice of perimenopausal syndrome, but there is no persuasive evidence of its effect on perimenopausal panic disorder. Hence, some sensible evidence of its effectiveness and safety in treatment of perimenopausal PD is urgently needed. Imaging techniques can provide an effective approach to study the functional brain region. With the help of fMRI and 1H-MRS, the changes in neural activity before and after the study can be gathered. Thus, pathogenesis of perimenopausal PD may be revealed as well as the neural mechanism of acupoint catgut embedding treatment.

## 2 Methods/design

### 2.1 Trial design

This is a parallel designed, exploratory randomized controlled trial with a 1:1 allocation to the intervention group (Group Embedding, n = 35) and the control group (Group Medication, n = 35). The study is intended to investigate the effectiveness and safety of acupoint catgut embedding for perimenopausal PD. The other objective is to explore the central nervous mechanism of this disease, to research the internal relationship between neural activity and amino acid metabolism with the use of fMRI and 1H-MRS. The protocol adheres to the SPIRIT 2013 statement. The trial will be done in the the Bao’an District TCM Hospital, the Affiliated Hospital of Guangzhou University of Chinese Medicine.

### 2.2 Study subject

All the subjects are menopausal syndrome specialist clinic patients from the perimenopausal syndrome clinical research base of Guangdong Provincial Hospital of Traditional Chinese Medicine and the Bao’an District TCM Hospital, the Affiliated Hospital of Guangzhou University of Chinese Medicine.

### 2.3 Recruitment

There are 3 ways to recruit participants: ① Recruit from clinic patients of cooperative hospitals. We will gather each hospital's director of acupuncture department and gynecology department and call a seminar focusing on how to recruit participants. Then we will send assistants to each department to assist screen participants. Clinical trial information will be posted on the bulletin boards of these hospitals. ② Recruit via broadcasting, TV and newspapers, etc. ③ Attract suitable participants by introducing some basic knowledge about perimenopausal physiology, perimenopausal pathology and its treatment in community service.

### 2.4 Eligibility criteria: inclusion criteria

1. Corresponding to diagnosis standards of PD;
2. Female, aged 41 to 60, at least junior middle school literacy;
3. HAMA score ≥ 14;
4. Patients who consent to sign informed consent form.

### 2.5 Eligibility criteria: exclusion criteria

1. Combined with other mental disorders, such as phobia and melancholia, or PD caused by somatoform disorders;
2. Combined with severe cardiac and hepatic and renal insufficiency, or other serious diseases of other systems like malignant tumors;
3. PD caused by physical diseases like epilepsy, heart attack, pheochromocytoma, hyperthyroidism, or spontaneous hypoglycemia, etc;
4. Patients who used antipsychotic drugs, antidepressive drugs, or sex hormones within 2 weeks before included, or combined taking other therapies like cognitive behavioral therapy etc;
5. Patients who have contraindications to MR, like having aneurismal clips, implantable neuro-stimulator, pacemaker, implantable automatic defibrillator, cochlear implantation or visual obstacles;
6. Female during pregnancy or lactation;
7. Patients who suffered from alcohol or drug abuse within 1 year or with a history of alcohol or drug dependency.

### 2.6 Subject withdrawal criteria

1. Violation of the inclusion criteria or fulfillment of the exclusion criteria;
2. Serious adverse events occur during the treatment;
3. Violation of the protocol by the investigator or the subject;
4. Use other medicines or treatments without permission from the investigator;
5. Loss to follow-up period;
6. Inappropriate progress as judged by the investigator.

### 2.7 Randomization

Random numbers will be generated by PEMS 3.1, and made into random cards. Then the cards will be sealed in opaque envelopes, kept by designated personnel. There is a one-to-one correspondence between the registration of clinic patients and the serial number of the envelope which contains the random number. Based on the number of the envelope each patient gets, these 70 patients will be grouped into the intervention group and the control group at random, and each group will contain 35 samples.

### 2.8 Blinding

Since it is unachievable to apply blind method to experimental subjects of the two groups above and the experimenters, result measurers and data statisticians will be blinded.

### 2.9 Intervention

At the screening visit, participants will sign the informed consent form. In a baseline questionnaire, information will be obtained age, occupation (physical/non-physical), education level, last menstrual period, previous use of acupuncture and expectation to acupoint catgut embedding therapy. Moreover, Panic Disorder Severity Score (PDSS), Panic-associated Symptoms Score (PASS), Hamilton Anxiety Scale (HAMA), Symptom Checklist 90 (SCL-90) must be completed at the baseline (week 0). The frequency of panic attack will also be recorded. Thereafter, study subjects will be randomized. There is a 3-months study period and a 1-month follow-up period for each group. In the study period, the frequency of panic attack, PDSS, PASS, HAMA Score and SCL-90 Score will be evaluated every 4 weeks. fMRI and 1H-MRS will be tested two times: in week 0 and week 12. A detailed process flow according to the SPIRIT figure can be seen in Table 1.

**Table 1:**
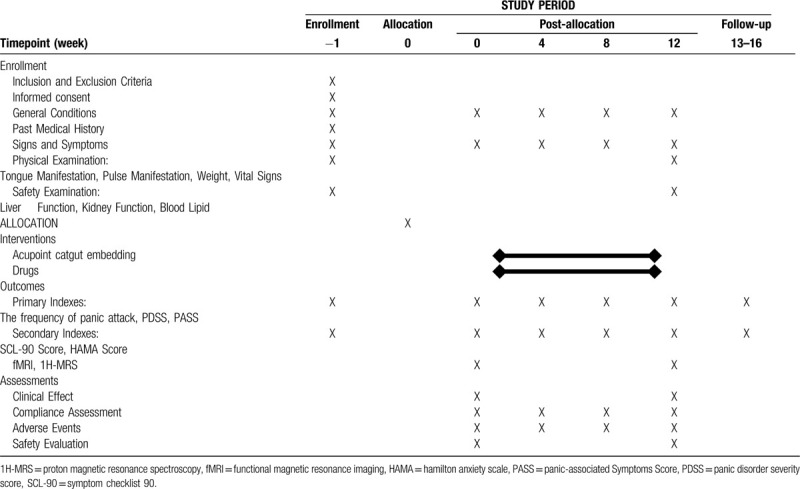
Schedule of enrollment, interventions and assessments.

#### 2.9.1 Intervention group

Acupoint catgut embedding therapy will be applied to patients in the intervention group fortnightly, a total of 6 times during 3 months. Main acupoints are BL23, SP6 and RN4. If it is a liver-kidney yin deficiency case, BL18 will be added. If it is a kidney yang deficiency case, ST36 and BL20 will be included. In each treatment, RN4 is a required acupoint. The rest acupoints can be used in the alternation of left/right side. The location method is according to *Location of Acupoints* the National Standards of P.R promulgated by the State Bureau of Technology Supervision. The operating method follows the National Standards of P.R (GB/T 21709.10-2008) manipulations of acupuncture and moxibustion—Part 10 Acupoint catgut embedding. Details about the intervention group interventions are described in the Standards for Reporting Interventions in Clinical Trials of Acupuncture (STRICTA) checklist in Table 2.^[14]^

**Table 2:**
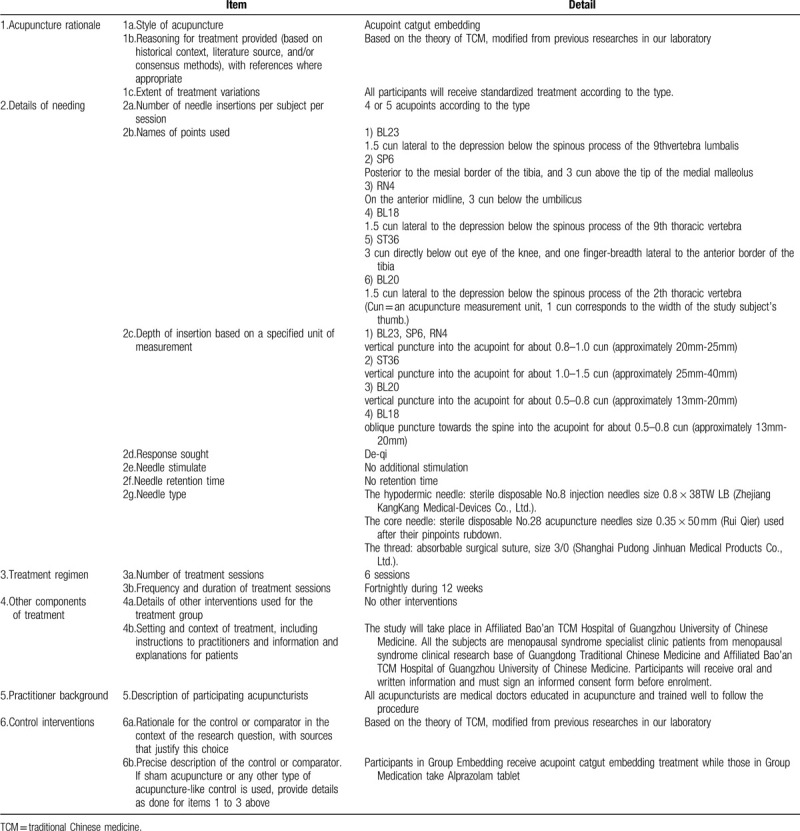
Details of acupuncture treatment for perimenopausal panic disorder, based on the STRICTA 2010 checklist.

#### 2.9.2 Control group

In the 3-months study period, patients in the control group will take Alprazolam tablet (Guangxi Southpearl Pharmaceutical Co., Ltd.) with the size of 0.4 mg orally, 1 tablet a time, 3 times a day.

#### 2.9.3 fMR examination procedure

The fMRI (PRISMA 3.0T Magnetic Resonance Imaging Scan; Siemens Company) examination will be performed to collect brain images offering more visualized proof. During the scanning procedure, subjects will be asked to keep quiet and their eyes closed. In the meantime, they need to empty their minds but avoid sleeping. The researcher will check whether the participants are awake during the process. For T1-weighted images (Spin Echo) scan, parameters are TR/TE = 2100 ms/24 ms, field of view = 240 mm × 240 mm, matrix size = 256mm × 256 mm, voxel size = 3 × 3 × 3mm^3^, 18 sections, 5 mm thickness, 1.5 mm slice gap, 20.83 Hz/pixel bandwidth. DTI images (SE EPI), in transaxial plane aligned to the anterior and posterior commissure line, are acquired with the following parameters: matrix size = 128mm × 28 mm, field of view = 240mm × 240 mm, 3.8 mm slice thickness, 28 slices centered on the corpus callosum, b-value = 1000 s/mm^2^. The parameters of T2-weighted images (Gradient-Echo, Echo Planar imaging, GRE-EPI) are TR/TE/FA = 3000ms/60ms/90°, field of view = 240mm × 240 mm, matrix size = 64mm × 64 mm, 18 sections, 5 mm thickness. The data acquisition is carried out 12 seconds after the start of scanning, and the scanning time is 5 minutes and 12 seconds. The blood oxygen level dependent (BOLD) signal imaging sequence is monitored by RTIP.

#### 2.9.4 1H-MRS examination procedure

1H-MRS data are acquired with GE EXCITE Signa 3.0T MR. Structural images for orientation using the following parameters: the axial and coronal plane, TR/TE = 3647 ms/100 ms, 1 mm thickness, no slice gap. 1H-MRS voxels are positioned in the Hypothalamus (1.5 × 1.5 × 1.0 cm). Spectra are acquired using Point Resolved Spectroscopy (TE = 144 ms; TR = 1500 ms; NEX8) and analyzed using SPM8 software provided by the Philips Company. The position of N-acetyl-aspartate (NAA) is determined at 2.0 ppm on the spectral line, and the chemical shifts of other substances are determined with NAA as reference. So Glutamic acid complex (Glx, Glu + Gln) is located at 3.8 ppm, GABA is located at 3.0 ppm, acetylcholine complex (Cho) is located at 3.2 ppm, creatine (Cr) is located at 3.02 ppm. The area under each peak is measured in the calculation. The ratio of NAA, Cho to Cr is calculated with Cr as the internal standard.

### 2.10 Outcome measures

#### 2.10.1 Primary endpoint

The primary outcomes of this study will be changes in the frequency of panic attack and in the PDSS and PASS during the observation period (3 months) and follow-up period (1 month), analyzed in both groups. After baseline time, they will be tested at week 0, week 4, week 8 and week 12, and during the follow-up period.

#### 2.10.2 Secondary endpoint

HAMA Score and SCL-90 Score will be assessed as secondary outcomes. HAMA Score is an essential diagnostic tool for anxiety disorders and is commonly used in clinical diagnosis and classification of anxiety disorders. SCL-90 Score has advantages of large capacity, rich symptoms, and more accurate characterization of patient's conscious symptoms. It can show mental status of the interviewee which is closely related to the quality of life. They will be tested at week 0, week 4, week 8 and week 12, and during the follow-up period.

#### 2.10.3 fMRI and 1H-MRS

fMRI will focus on the activity of the amygdaloid nucleus and the connection between the fear center and fear network to reflect the changes of the brain's function of perimenopausal PD patients before and after treatment. 1H-MRS will test the Glutamate-GABA level of these brain tissues to explore the relations between neural activity and amino acid metabolism. Differences in imaging changes will be compelling evidence.

### 2.11 Safety assessment

At every visit, physical examination will be performed, and adverse events will be checked. Blood samples will be collected at week 0 and week 12. Blood routine test, liver function (Alanine aminotransferase) and kidney function (serum creatinine) will be done at the beginning and the end of the intervention period.

### 2.12 Adverse events

In research, some adverse reactions may occur.

1. There are a few common responses after the acupoint catgut embedding:
  - Local reactions: Mostly some sterile inflammations with inflamed hot pain occur within 5 days. Some cases may be more severe, like a small amount of milky-white exudation at the embedding spot caused by fat liquefaction because of suture stimulation. Circumstances above should not require any special treatment.
  - General reactions: Some patients may experience temperature rising after treatment 4 to 24 hours, mostly around 38. It will fade away in 2 to 4 days by itself. Usually, hemogram of each patient may rise in different levels, and it will return to normal in 3 to 5 days.
2. There are a few adverse responses after the acupoint catgut embedding:
  - Pain: Apply hot compress if there is pain at embedding acupoint.
  - Secondary infections: Lack of strict asepsis and inappropriate protection for the wound could cause secondary infection, which mostly brings about some inflammatory symptoms like local swelling, progressive pain, and fever in 3 to 4 days after the implantation. Hot compress and anti-infective treatment would be helpful.
  - Nerve injuries: It is usually caused by an incorrect operation or excessive stimulation or carelessness, and can be avoided with careful operation.
  - Hemorrhage: It is usually caused by a puncture on the vessels or excessive stimulation. Pressure put on the puncture point would stop the bleeding.
  - Suture allergies: Local itching or swelling or fever can be remitted by antianaphylaxis treatment.
3. Alprazolam is one of the Benzodiazepines. Its principal effects are anti-anxiety and antidepressant. Common adverse reactions are as following:
  - Somnolence, vertigo, and weakness, etc. The large dose may cause dystaxia, tremor, uroschesis or jaundice.
  - Addiction. Drug withdrawal may cause withdrawal symptoms after being used for a long period, showing as emotional or melancholy.
  - A few patients may suffer dry mouth, inattention, hyperhidrosis, palpitation, constipation or diarrhea, blurred vision, and hypotension.

### 2.13 Sample size calculations

Based on preliminary experiments, it is obtained that after treatment the Kuppermann index (MI) of the intervention group dropped 15.90 ± 2.71, while the control group dropped 13.84 ± 1.87, the population standard deviation S = 2.12, δ = 2.06. Given α = 0.05 bilateral, β = 0.1, power of test 1−β = 0.9, based on formula^[15]^: n = 2 × [(tα+t2β) × s/δ] × [(tα+t2β) × s/δ], calculating with trial and error method, we used table look-up: tα, ∞ = 1.96, t2β, ∞ = 1.282, then we obtained n = 23.66 ≈ 24. According to t table: t0.05(24 × 2−2) = 2.0129, t0.2(24 × 2−2) = 1.3002, we substituted it into the former formula and obtain n = 23.25 ≈ 24, the sample size was plateaued, and the attempts stopped, which means each group requires 24 samples, and 2 groups require 48 samples in total (n = 48). With the consideration of the maximum loss rate of follow up is 20%, we concluded that the total sample size is 60. According to HAMA Score (14 items contained), the sample size is 14 × 5 = 70. Take two parts above into consideration, the sample size is determined at 70.

### 2.14 Statistical analysis

Image data processing: using methods like independent component analysis (ICA) and function binding, applying professional software such as MRIcro for windows, Xjview 8.1, SPM 12 and MATLAB 5.1.

Statistical analysis technique: to apply a statistical test to the difference of prior-treatment and post-treatment, and to examine the curative effect difference within groups and among groups of all curative effect indexes. We will adopt the two-sided test for all statistical tests. It's considered that the tested differences are statistically significant if *P* less than or equal to 0.05. If *P* greater than or equal to .05, the tested differences are not statistically significant. Details are as following.

Measurement data: The *t* test will be adapted to run comparison among groups. When it doesn’t comply with the normal distribution, we will choose Wilcoxon rank-sum test. Similarly, we will run paired *t* test towards the difference of prior-treatment and post-treatment, and it will be changed into Wilcoxon rank-sum test while it does not comply with the normal distribution.

Enumeration data: We will use the Chi-square test, calibration Chi-square test, and Fisher exact method to run comparison among groups.

Ranked data: Wilcoxon rank-sum test will be applied to run comparison among groups and signed rank-sum test to run comparison within groups.

### 2.15 Quality control

All the investigators have been trained well to follow the trial's procedure and acupuncturists are instructed to give acupuncture treatment merely. Any other treatment or counseling is forbidden. To ensure the quality of the study, clinical monitors will arrange specialized staff to check the process of the trial and the details of medicine use. Additionally, monitors nominated by the principal investigator will verify the accuracy and validity of the original data which should be uploaded in time and accompanied with text summaries. Regular meetings will be organized to handle the difficulties and problems emerging during the study.

## 3 Discussion

PD is also known as the acute anxiety attack (AAA), one of the main patterns of manifestation of anxiety disorder.^[16]^ Repeated outbreaks of panic, tachypnea, dizziness, palpitation, tremor, and sweating are its common symptoms. Modern medicine towards perimenopausal PD has a bunch of disadvantages, for example, hormone replacement therapy could increase the risk of higher morbidity of endometrial cancer, breast cancer, and colorectal cancer. Non-hormonal medication therapy may lead to nausea and vomiting, dizziness and headache, weakness and tremor, the change of weight. More importantly, low efficiency and high relapse are the biggest problems. In recent years, therapy combining CBT with pharmacotherapy has become a hot research area of treating PD. However, it still cannot solve these problems. These existing treatments have either little effectiveness or many side effects. Because many patients are dissatisfied with current treatments, the demand for acupoint catgut embedding is increasing. ‘Keep it for a long time to cure chronic diseases’ is the theoretical guidance of acupoint catgut embedding therapy. Based on traditional acupuncture technique, this therapy is developed by using modern technology. It takes advantage of the stimulation of acupoint via physicochemical change of the suture in the human body. It is a comprehensive regulative process. However, well-designed clinical trials on the effectiveness and safety of this therapy for perimenopausal PD are not available.

This study aims at proving the clinical effect and safety of acupoint catgut embedding. Meanwhile, with the help of fMRI and 1H-MRS, investigating the pathogenesis and physio-pathology characters by revealing neural activity and amino acid metabolism of perimenopausal PD patients is a significant goal as well. There are many studies showing different mechanisms in this disease. Our laboratory runs a variety of researches aiming at the GAMA mechanism in long terms. In this mechanism, free amino acid is an iconic index. And study has also shown the changes in fMRI images of perimenopausal PD patients.

Based on structural data from human and animal researches, brain-imaging studies have identified a network of structures that constitute the neural circuitry for emotions, including the amygdala, cingulate, insula and prefrontal cortex. They interact to distinguish the emotional significance of stimulations, to generate and regulate affective states further.^[17,18]^ Amygdaloid nucleus is described as the “fear center” of the brain or the core of “fear network”.^[19]^ Studies have shown that PD patients have an excessive activity of the amygdaloid nucleus and Hypothalamus-Pituitary-Adrenal axis (H-P-A axis). With the improvement of the disease, the activity of amygdaloid nucleus tends to be normal. The prefrontal cortex participates in emotional processing. Anterior cingulate cortex is closely related to mood and self-control.^[20]^ As the connecting hub, insular lobe connects with the anterior cingulate cortex, amygdaloid nucleus, prefrontal cortex and other tissues which also form the circuit. Insula hyperreactivity is associated with processing negative emotional information,^[21–23]^ threat-relevant cues and symptom severity.^[24]^ Structural phase studies found abnormal volume of insula gray matter, anterior cingulate cortex and amygdaloid nucleus occur in PD patients. Functional phase studies revealed that abnormal activities of amygdaloid nucleus, anterior cingulate cortex and prefrontal cortex lead to cognitive loss and predictive anxiety,^[25,26]^ suggesting the close connection between the occurrence and development of PD and neuro-anatomic structures.

In neuroimaging researches with CBT therapy, the most consistent findings regarding response to treatment were observed in the amygdala, insula, hippocampus and anterior cingulate, so they can be seen as relevant predictors of treatment response in anxiety disorders.^[27]^ After successful treatment with CBT, changes of brain activation in cortico-limbic structures have been discovered, particularly a inhibitory functional coupling between anterior cingulate cortex and amygdala concerning the response to treatment.^[28]^ Studies have shown that the precuneus and amygdaloid nucleus in patients with PD are related to anterior cingulate and negatively correlated with γ-GABA levels in the anterior cingulate.^[29]^ Neurological imaging studies have shown that Glutamate and GABA are the most important amino acid neurotransmitters, maintaining brain's excitement and inhibition, mediating conduction of more than 90% of brain's nerve. Combining with the outstanding disruptions in amino acid metabolism of OVX rats,^[13]^ we hypothesize that the metabolic disorder of amino acid, more accurately the imbalance of Glutamate-GABA caused by GABA's content decreasing, causing the excitatory and inhibitory imbalance of brain function, maybe the main etiological agent of perimenopausal PD.^[30]^

Early studies have shown the advantage of our study that acupoint catgut embedding has a significant effect on perimenopausal PD without side effects.^[31,32]^ Many previous studies are lack of enough indexes to reveal and measure the functions of the treatment.^[33,34]^ But our study will include credible scales and imaging changes of fMRI and 1H-MRS as persuasive evidence. Unfortunately, at present the syndrome differentiation of TCM is not certain yet. Diagnostic criteria and curative effect evaluation have no unified standard. All these factors restrict our research.

In conclusion, the results of this trial are expected to provide evidence for the effectiveness and brain correlates of acupoint catgut embedding treatment for perimenopausal PD. To reveal the pathogenesis and physio-pathology characters of perimenopausal PD with the help of fMRI and 1H-MRS is another expectation. We believe the outcome will exert a positive effect on acupoint catgut embedding treatment for perimenopausal PD.

### 3.1 Trial status

Version number of the protocol: 15010000002006495

Recruitment for the trial started in December 2017 and is expected to end in December 2021.

## Author contributions

GZC conceptualized the study and drafted and revised the manuscript. YXX contributed to the development and refinement of the study protocol. XW and SZ drafted and revised the manuscript. XKX and JQL made contributions to the neuroimage methods and statistical analysis. All authors reviewed and approved the final manuscript.
